# Genomic Approaches to Study Genetic and Environmental Influences on Fish Sex Determination and Differentiation

**DOI:** 10.1007/s10126-012-9445-4

**Published:** 2012-04-29

**Authors:** Francesc Piferrer, Laia Ribas, Noelia Díaz

**Affiliations:** Institut de Ciències del Mar, Consejo Superior de Investigaciones Científicas (CSIC), Passeig Marítim, 37-49, Barcelona, 08003 Spain

**Keywords:** Gonads, Teleost, NGS, Epigenetics, Microarrays

## Abstract

The embryonic gonad is the only organ that takes two mutually exclusive differentiating pathways and hence gives rise to two different adult organs: testes or ovaries. The recent application of genomic tools including microarrays, next-generation sequencing approaches, and epigenetics can significantly contribute to decipher the molecular mechanisms involved in the processes of sex determination and sex differentiation. However, in fish, these studies are complicated by the fact that these processes depend, perhaps to a larger extent when compared to other vertebrates, on the interplay of genetic and environmental influences. Here, we review the advances made so far, taking into account different experimental approaches, and illustrate some technical complications deriving from the fact that as development progresses it becomes more and more difficult to distinguish whether changes in gene expression or DNA methylation patterns are the cause or the consequence of such developmental events. Finally, we suggest some avenues for further research in both model fish species and fish species facing specific problems within an aquaculture context.

## Reproduction-Related Problems in Finfish Aquaculture

**Fig. 1:**
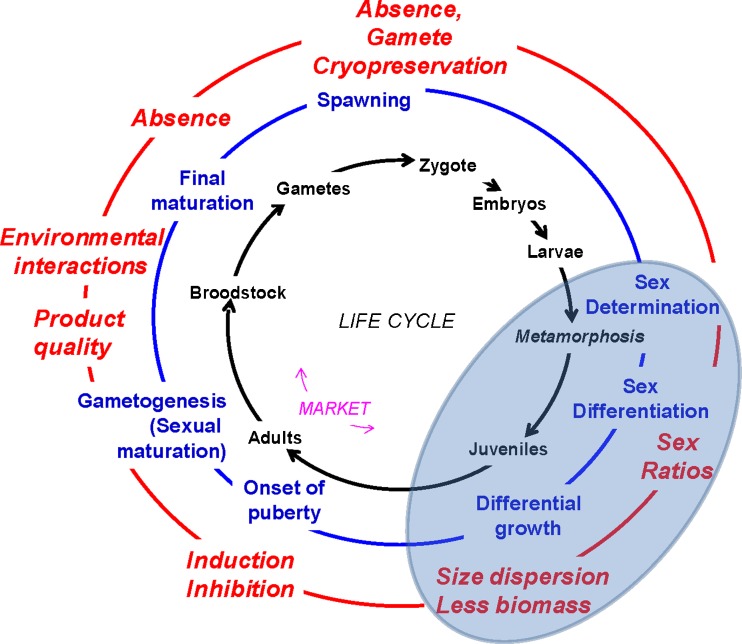
Reproduction-related problems in finfish aquaculture. The *inner circle* shows the different stages of the typical life cycle of finfish (not referred to any particular species), starting clockwise with the zygote, and indicating the approximate stage when marketing takes place. The *middle circle* shows events related to reproduction, starting with sex determination and finishing with spawning. The *outer circle* shows typical reproduction-related problems in aquaculture, linked to the former reproductive events (see text for further explanation). The *shaded area* represents the issues dealt with in this paper

The existence of reproduction-related problems in current finfish aquaculture depends upon the species considered. The main problems that fish may experience under captive conditions are skewed sex ratios, the existence of sexual growth dimorphism, the lack of sexual maturation or side effects of it on product quality, especially if maturation occurs before marketing, and the absence of final maturation or of gamete release (Fig. 1).

Under culture conditions, some species either do not reproduce altogether or exhibit sex-related problems in reproduction. This is, for example, the case of the Senegalese sole (*Solea senegalensis*), where F1 males, which are normally oligospermic, do not produce or release sperm (Cerdà et al. 2008), a situation that has significantly hampered the development of the aquaculture for this species. On the other hand, and as it is part of the biology of many fish (Breder and Rosen 1966; Parker 1992), in many aquacultured species one sex grows more than the other does. This sexual growth dimorphism can favor males (e.g., tilapias) or, more commonly, females (flatfishes, sea basses, etc.). In some cases, as for example in the turbot (*Scophthalmus maximus*), females can be up to 50 % larger than males (Imsland et al. 1997). In other cases, as in European sea bass (*Dicentrarchus labrax*), the rearing conditions result in highly male-biased stocks (Piferrer et al. 2005), and if males, as it happens also to be the case in this species, grow less than females, then the exploitation is ran at a suboptimal capacity. Thus, skewed sex ratios induced by the captive conditions in a particular species may have negative consequences because of the sex growth dimorphism stated earlier. It is then clear that knowing how sex ratios are established would contribute to the development of methods to achieve monosex populations consisting only of the desired sex. A great deal of research towards the development of such sex control methods has been carried out in fish (Piferrer 2001). Finally, many species of fish also mature precociously when subjected to the fast growing conditions of modern aquaculture. This affects particularly males, and a well-known example also concerns the European sea bass (Felip et al. 2006). In other cases, such as in the rainbow trout (*Onchorhynchus mykiss*), monosex stocks are desired not as much because one sex grows more than the other, as is also the case in many salmonids, but because maturation affects the organoleptic properties of the edible parts more in one sex than in the other. Therefore, female monosexing in salmonids is especially useful when associated with triploidy because triploid females do not develop ovaries while triploid males still are able to develop testis (Piferrer et al. 2009). In addition, in species such as the sturgeons, the advantage of getting only females is due to the value of the ovaries as a source of caviar. Thus, knowing the underlying molecular mechanisms present along the brain–hypophysis–gonadal axis would also help in devising methods to alleviate maturation, including precocious maturation. In addition, in newly aquacultured species such as the bluefin tuna (*Thynnus thunnus*), many aspects of reproduction are still not known and therefore studies on the reproductive of this and other potential species for aquaculture are needed (e.g., Aranda et al. 2011) .

The physiology, including the reproductive physiology, of an animal can be monitored largely not only by measuring key hormones but also by examining the expression of individual genes or a set of genes. Traditionally, this has been achieved in what is known as the “candidate gene approach”. Recently, with the advent of genomics, a range of new possibilities have emerged where one can examine the expression of thousands of genes and gene networks at once, contributing to a better understanding of key signaling and regulatory pathways related to important biological functions such as reproduction. The moment has arrived when this knowledge can be channeled to applied methods within an aquaculture production context.

This paper briefly reviews the application of genomic approaches to the study of reproduction in fish of relevance for aquaculture, focusing on studies aimed at improving our understanding of the complexities of gene expression patterns underlying the establishment of sex ratios.

## Background on Fish Sex Determination and Differentiation

**Fig. 2:**
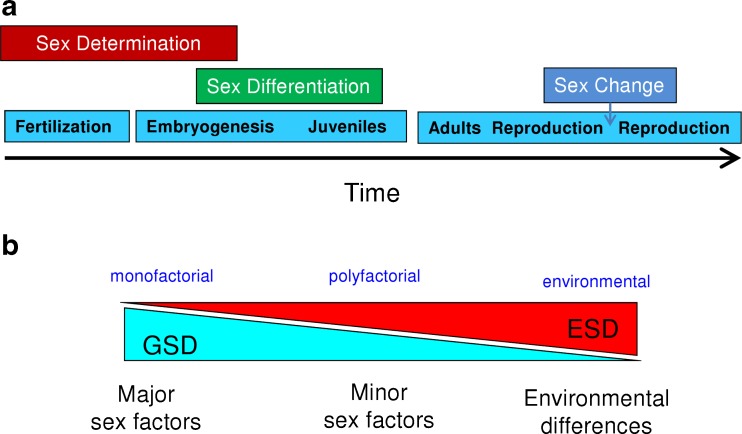
Sex determination and differentiation in fish. **a** The processes of sex determination, sex differentiation, and sex change are represented along the timeline of development. Sex change only occurs in sequential (a.k.a. consecutive) hermaphrodites. **b** The three major components of fish sex determination are indicated *below*. Genotypic sex determination (GSD) is driven by major and/or minor sex factors and environmental sex determination (ESD) is driven by environmental differences. The diagram also indicates *above* three common sex determination mechanisms

Sex ratios are an important aspect of populations because these not only determine their reproductive potential but also directly influence growth dynamics, and this is very important in farmed animals and thus relevant in finfish aquaculture. The sex ratio is the product of sex determination, the genetic and/or environmental process that establishes the gender of an organism (Penman and Piferrer 2008), and of sex differentiation, the various genetic, physiological processes that transform an undifferentiated gonad into a testis or an ovary (Piferrer and Guiguen 2008) (Fig. 2a). Sex determination in fish can range from genotypic (genotypic sex determination, GSD) to environmental sex determination (ESD), with temperature-dependent sex determination (TSD) being the most common type of ESD (Fig. 2b). Controversy has surrounded the issue of the abundance of TSD species, and in fact species that exhibit TSD under natural conditions of temperature are less common than initially thought (Ospina-Alvarez and Piferrer 2008), although most TSD species have no aquaculture or fishery potential, with few notable exceptions such as the pejerrey (*Odontesthes bonariensis*). Reasons for the comparatively low abundance of TSD vs. GSD species have been recently demonstrated based on model simulations (Grossen et al. 2010). Nevertheless, under some conditions, including often the ones present in fish farms, many GSD species are capable of responding to the influence of the environment (notably, temperature effects, TE), exhibiting biased sex ratios. The GSD species in which sex ratios are established by a combination of genetic and environmental influences are referred to as GSD + TE species. Examples of GSD species include the salmonids, halibut, and turbot, whereas GSD + TE species include European sea bass and Southern flounder (*Paralychthys olivaceus*). It should be noted that this classification serves only to group species according to their typical response to environmental changes, temperature in this case, but the continuum between GSD and TSD exists. Thus, even GSD species like salmonids may show a few strains able to respond to the influence of the environment (Magerhans and Hörstgen-Schwark 2010).

In GSD, the establishment of sex is based on the inheritance of major or minor sex factors. The former, also known as chromosomal sex determination, include monofactorial and multifactorial sex-determining mechanisms, with the presence of a single pair of sex chromosomes or multiple sex chromosomes. Importantly, in fish, both male (as in mammals) and female (as in birds) heterogamety exist. Furthermore, sex chromosomes can be homomorphic or heteromorphic (see Table 1 of Penman and Piferrer (2008) for a complete classification of GSD and TSD mechanisms). Thus, in fish, the sex-determining mechanisms are diverse and often different even in closely related species (Tanaka et al. 2007). This great plasticity of the sex-determining mechanisms has been linked to the great diversity of species in fish since plasticity in sex determination favors speciation (Qvarnström and Bailey 2009). In this context, it is not surprising that, because of such diversity of sex-determining mechanisms, the isolation of master sex-determining genes in fish is so far limited to the *dmy* gene of the medaka, *Oryzias latipes* (Matsuda et al. 2002; Nanda et al. 2002). However, since sex-linked markers and/or the sex-determining region is known already in several species, including aquaculture-relevant species such as the catfish—*Clarias gariepinus* (Kovacs et al. 2000), Nile tilapia—*Oreochromis niloticus* (Ezaz et al. 2004), rainbow trout (Felip et al. 2005), turbot (Martínez et al. 2009), and the half-smooth tongue sole—*Hippoglossus stenolepis* (Shao et al. 2010), the application of NGS (Next Generation Sequencing) techniques will help in the eventual identification of the sex-determining genes.

In contrast with the situation with sex determination, the genes and gene networks implicated in fish sex differentiation appear to be quite conserved throughout the species and even across the different groups of vertebrates (Piferrer 2011; Herpin and Schartl 2011; Munger et al. 2009). As in the rest of vertebrates, fish gonads have two major functions: gametogenesis and steroidogenesis. As a gland, the major products of synthesis of the gonads are the sex steroids, androgens (mainly testosterone, 11ß-hydroxyandrostenedione and 11-ketotestosterone), and estrogens (mainly estradiol-17ß). Androgens and estrogens are implicated in male and female sex differentiation, respectively. However, while estrogens are required (i.e., are the cause) for the differentiation and maintenance of the female phenotype, androgens, in contrast, are currently viewed as the consequence of male differentiation. The gonads also produce progestagens, but there is no evidence of their implication in sexual differentiation. Thus, the major products of synthesis of the adult gonads, sex steroids, are also involved in their differentiation. The genes implicated on the formation and the differentiation of the gonads can be grouped into three broad classes: those that are implicated in organogenesis (and thus ubiquitous in the formation and differentiation of many organs of the body), those usually more expressed during male differentiation, and those more expressed during female differentiation. Notice that many genes are present during both male and female sex differentiation, the difference being only the degree of expression levels. In addition, most of the genes better studied during sex differentiation fall in one of the following categories: steroidogenic enzymes, sex steroid receptors, transcription factors, and growth factors, but there are others. A description of these genes can be found in Piferrer and Guiguen (2008). Among these genes, the steroidogenic enzyme gonadal aromatase (*cyp19a1*) and the transcription factor *dmrt1* occupy a prominent place. Aromatase is solely responsible for the irreversible conversion of androgens into estrogens and thus determines the balance between these two antagonistic sex steroid types. Currently, it is thought that in most non-mammalian vertebrates, female sex differentiation depends essentially on the stimulation of *cyp19a1* through a positive feedback loop involving the transcription factor *foxl2*, while male sex differentiation would follow the inhibition of *cyp19a1* expression (Guiguen et al. 2010). Inhibition of *cyp19a1* would be achieved by *dmrt1* upregulation. This transcription factor is well conserved across taxa and has been shown to be a proven inhibitor of *cyp19a1* in fish (Wang et al. 2010). Upregulation of *dmrt1*, in turn, would be achieved by different mechanisms such as high temperature in a TSD or GSD + TE species, a male master sex-determining gene or an associated gene such as *sox9* in a GSD species with a monofactorial system of sex determination, or by the additive effects of several male-promoting genes in a polyfactorial system.

The conserved role of aromatase in female sex differentiation in fish (reviewed in Guiguen et al. (2010)) is exemplified with the fact that the effects of temperature on sex ratios are invariably linked to an inhibition of *cyp19a1* expression. This has been observed in species with a strong genetic basis of sex determination (pure GSD species) such as the Atlantic halibut, *Hippoglossus hippoglossus* (van Nes and Andersen 2006), in species with a combination of genetic and environmental sex determination (GSD + TE species) such as the olive flounder (Kitano et al. 1999), or in species with true temperature-dependent sex determination (TSD species) such as the pejerrey (Karube et al. 2007).

## Approaches to Study Genomics of Fish Sex Determination and Differentiation

There are several approaches to study genomics of fish sex determination and differentiation. This issue has been discussed in some detail elsewhere (Piferrer and Guiguen 2008). A brief update is discussed in the following text.

As stated above, the existence of several sex-associated markers can facilitate the search for the sex-determining region or sex-determining gene(s). However, due to the low morphological differentiation of sex chromosomes, large differentiated genomic regions are uncommon in fish. Thus, screening these regions has not always been successful as opposed to the combined used of medium- to high-density genetic maps and carefully selected family crosses (Martínez et al. 2009; Shapiro et al. 2009). In addition, due to the good synteny between fish species, comparative genomics can provide useful information towards the localization of the sex-determining region or gene (Sarropoulou et al. 2008; Martínez et al. 2009).

**Fig. 3:**
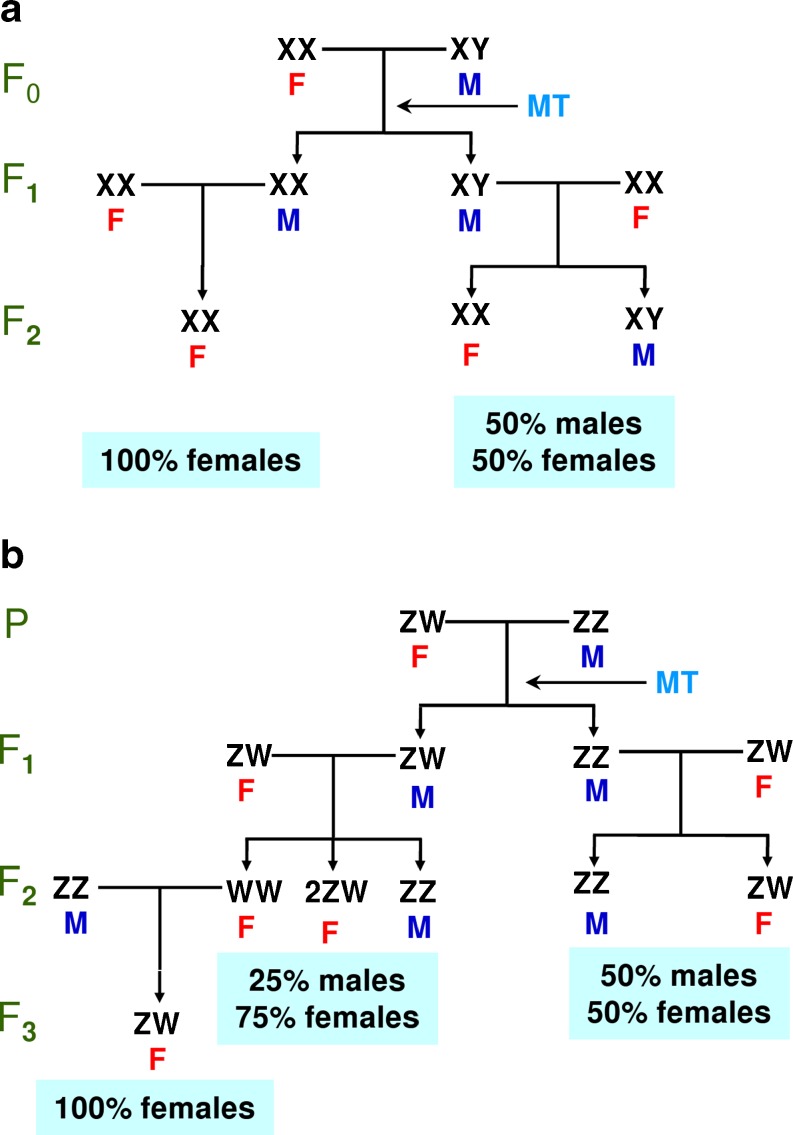
Methods to produce all-female stocks based on endocrine therapy. The indirect method of feminization by androgen treatment is shown for two types of species: **a** with female homogamety and **b** with male homogamety. In this case, the viability of the WW genotype is assumed. *M* male, *F* female, *MT* 17α-methyltestosterone

From the point of view of the biology of the species under study, the approach usually taken to study the genomics of fish sex determination and differentiation depends to a large extent on whether monosex populations are available. These monosex populations have been achieved in species with a XX/XY system of sex determination such as the rainbow trout or Nile tilapia (*Oreochromis niloticus*) by crossing normal females with sex-reversed genotypic females into phenotypic males with androgen treatment (Fig. 3a) (Piferrer 2001). The resulting progeny is all-female. In species with a ZW/ZZ system of sex determination, a similar approach is also feasible but it takes more time, even if fish with the WW genotype are viable (Fig. 3b). In some species, YY males (XX/XY system) can be obtained and are viable if the Y chromosome has not experienced too much degeneration, and they can be used to obtain all-male populations. Conversely, ZZ females (ZZ/ZW system) can also be used to get all-male populations. Finally, considering gynogenesis, which usually gives monosex female populations in the XX/XY system, it may also be interesting to study the genetic control of ovarian differentiation without paternal effects. These possibilities provide an excellent tool to study the expression of genes related to a particular sex because during development one can sample a population with a known sex well before the sexual differentiation of the gonads takes place.

Recently, with the identification of the sex-determining system (ZW/ZZ) of the turbot (Haffray et al. 2009) plus its sex-determining region (Martínez et al. 2009), it is expected that with appropriate endocrine therapy (Piferrer 2001) the possibility of producing monosex populations will soon be feasible.

**Fig. 4:**
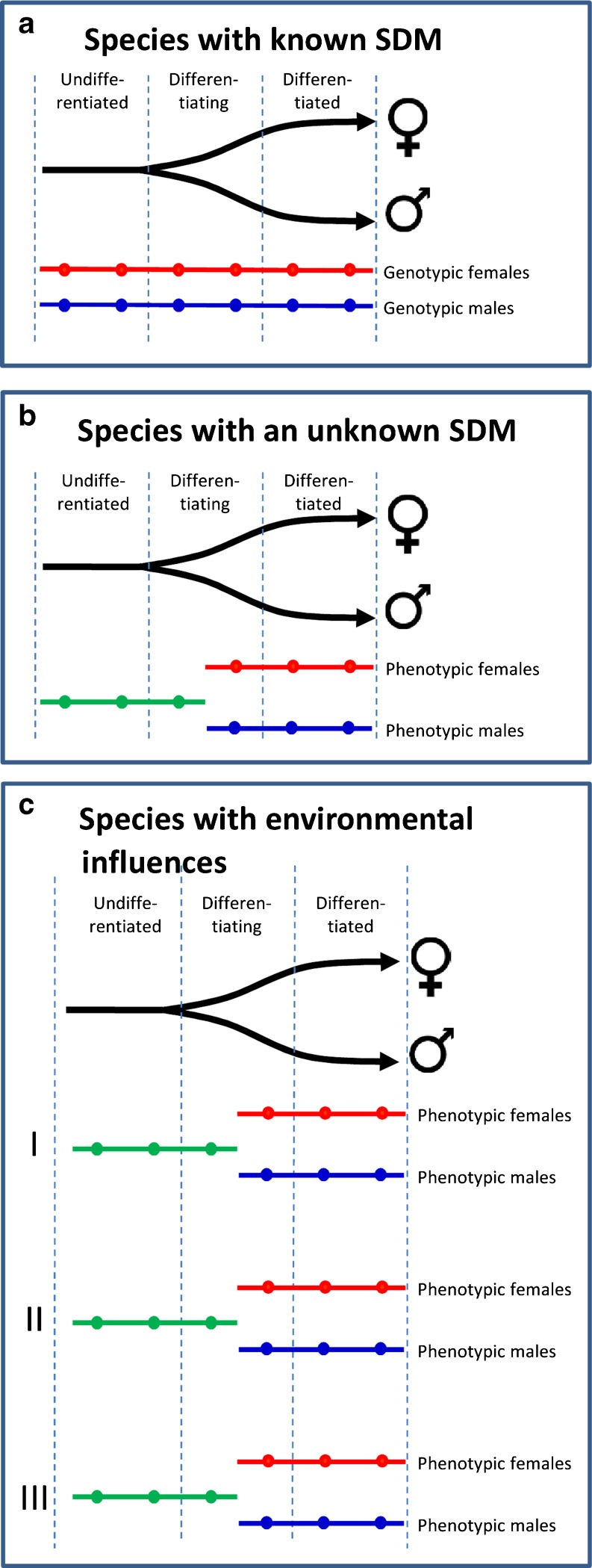
Strategies for the transcriptomic analysis of sex differentiation in fish. **a** Species with known sex determining mechanism (*SDM*), **b** species with an unknown SDM, and **c** species with environmental influences. RNA is harvested from genotypic females (*red line*), genotypic males (*blue line*), and undifferentiated fish (*green line*) at different times (*dots*) during development. In **c**, *I*, *II*, and *III* indicate that sampling has to be carried out including fish reared under different environmental conditions

Likewise, the strategies available for the transcriptomic study of the gonads during sex differentiation are also related to the type of sex-determining system. By virtue of the production of monosex populations, as described above, species with a simple XX/XY or ZW/ZZ sex-determining system are the most amenable to study since RNA samples can be obtained in populations of known genotypic sex not only after but, most importantly, during and even before the first signs of sex differentiation (Fig. 4a). In the rainbow trout, for example, this has enabled not only the identification of sex-related genes during sex differentiation but also the identification of a candidate gene for salmonid sex determination (Yano et al. 2011).

In species with (a) an unknown sex-determining system, (b) known sex-determining system but without monosex populations available, or (c) with sex-determining systems other than monofactorial, there is no other choice than to sample undifferentiated fish of unknown sex genotype and to compare gene expression profiles with those of differentiating males and females and with those of differentiated males and females (Fig. 4b). This strategy has been applied to study the gonadal transcriptome of males vs. females in the Senegalese sole (J. Viñas and F. Piferrer, unpublished observation) and in the turbot (L. Ribas, B. Pardo, C. Fernández, J.A. Álvarez-Dios, A. Gómez-Tato, J. Planas, P. Martínez and F. Piferrer). However, while this strategy is relevant in case of RNAs which can be extracted and analyzed in one individual, it can be problematic when samples obtained during the morphologically undifferentiated period consist of a mixture of gonads or body trunks from animals which can be developing males or developing females. This makes safe interpretation of data difficult, and the best one can hope is to refer to such type of mixtures to “undifferentiated fish or gonads” accepting these limitations. Thus, in these cases, sampling during this period of life raises specific questions and limits.

Finally, in species where sex determination is a combination of genetic and environmental influences such as European sea bass (polygenic mechanism with temperature influences; Vandeputte et al. 2007) or the pejerrey (TSD; Karube et al. 2007), a similar scenario than in the previous case emerges, but in order to obtain a reliable information on the interplay between genetics and environment, several samplings under different levels of the main or suspected environmental variable should be considered (Fig. 4c).

From the technical side point of view, it should be mentioned that until recently the study of sex determination and differentiation has been carried out by examining one or few genes in what is usually termed the “candidate gene approach”, i.e., the study, in a given species, of a gene or a set of genes known to play a certain role in other species, including phylogenetically distant ones. The aim is to find out if such a role was conserved or not. Since several years ago, however, research in this area has started to benefit from the development of custom-designed microarrays, which allow insight into signaling and gene regulatory pathways (e.g., Cerdà et al. 2008; Ferraresso et al. 2010), and, recently, of NGS technologies. These allow obtaining several millions of sequences, which can help in gene expression quantification (RNA-seq) and contribute to gene discovery. Table 1 summarizes some of the recent studies (last 5 years) on fish gonadal development using genomic approaches. These approaches allow the identification of tens of thousands of probes derived from gonadal tissues and comparison of the presence/absence or differing abundance of these probes between sexes. Interestingly, however, although such a large number of sequences are available, the number of specific or cognate genes involved in sex differentiation and determination processes can be several orders of magnitude lower. Thus, depending upon the study considered, it is not strange that the proportion of genes that are detected to be differentially expressed between sexes in the gonads may be as low as 0.01 % and usually <10 % of the total transcriptome of the organism. Thus, despite the availability of NGS technologies, studies concerning genes with a well-established role in sex determination–differentiation are still very much needed.

**Table 1:** Summary of some studies on fish gonadal development using genomic approaches

Species common name	Monosex	Stage	Method	Verification	References
Guppy	No	Adults	454 titanium	PCR and RNA-seq	Fraser et al. (2011)
Largemouth bass	No	Adults	GS-20 and microarray	qPCR	García-Reyero et al. (2008)
Rainbow trout	Yes	Juvenile	Macroarray, microarray	qPCR	Baron et al. (2007, 2008)
Senegalese sole	No	Adult female	Microarray	ISH and qPCR	Tingaud-Sequeira et al. (2009)
Sturgeon	No	Juvenile and adults	454 GS		Hale et al. (2009)
Sturgeon	No	Adults	454 GS and titanium	qPCR	Hale et al. (2010)
Nile tilapia	Yes	Larvae and fry	qPCR		Ijiri et al. (2008)
Nile tilapia	Yes	Adult	Cell transfections, transgenics	EMSA, ISH	Wang et al. (2010)
Pejerrey	No	Juveniles	Microarray	qPCR	Fernandino et al. (2011)
Platyfish	No	Adults	454 titanium	qPCR	Zhang et al. (2011)
Zebrafish	Transgenic	Fry	Transgenics	IHC, histology	Wang et al. (2007)
Zebrafish	No	Adults	Microarrays	ISH and qPCR	Sreenivasan et al. (2008)
Zebrafish	No	Adults	Microarrays	qPCR	Small et al. (2009)

454 titanium and 454 GS are commercial products from Roche

*EMSA* electrophoretic mobility shift assay, *GS-20* generation sequencing, *IHC* immunohistochemistry, *ISH* in situ hybridization, *qPCR* real-time quantitative PCR

In general, males seem to exhibit a more active transcriptome than females. Thanks to the gene ontology databases, some genes differentially expressed between sexes have been assigned a functional category. Although by no means what follows is intended to imply a generalization applicable to all species, based on the limited data available, so far it seems that, in general, genes of the female gonadal transcriptome commonly fall in the following categories: translation, regulation of transcription, meiosis, transport, angiogenesis, and antiapoptotic pathways. On the other hand, genes more expressed in males fall in the categories of development, signal transduction, translation, morphogenesis, cell communication, response to chemical stimulus, metabolic pathways, developmental processes, muscular contraction, and steroid biosynthesis.

**Fig. 5:**
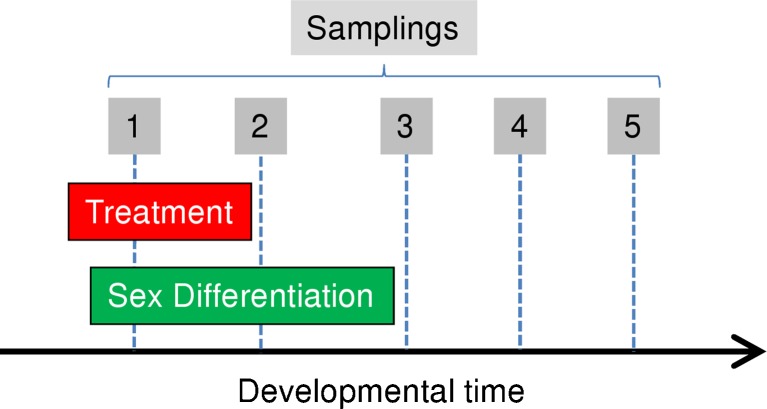
Effects of timing of RNA harvest in relation to the study of a developmental process such as sex differentiation. A treatment such as that with hormones, receptor blockers, or temperature is optional. *Boxed numbers 1* to *5* represent different time points where RNA is harvested: *1*, *2*, and *3* represent RNA harvest early, during, or at the end of the process being studied, while *4* and *5* represent later samplings

One aspect worth considering is the timing of RNA harvest in relation to the process being studied. Regardless of whether sex determination and differentiation are considered as two separate processes following a strict sequence or they are considered globally (e.g., Uller and Helanterä 2011), they are developmental processes that lead to the formation and differentiation of the gonads over a certain period. Thus, when investigating the genes implicated, one must ask whether observed changes in gene expression reflect the cause or the consequence of a given developmental pathway. This is especially relevant if a treatment (e.g., hormones, temperature) is applied in an attempt to determine the consequences of such treatment on the process. This is illustrated in Fig. 5. RNA harvest early during sex differentiation or while the treatment is being applied will measure immediate effects of the treatment, which may not be relevant in the long term for the question being asked. RNA harvest right at the end of treatment will provide information on the direct consequence of treatment. The disadvantage with such approach is that, because of the developmental dynamics of the gonads, the gonadal tissue will probably be dissected out along with other non-gonadal tissues, which compromises specificity. One way to alleviate this problem is by using germ cells labeled with GFP to aid in the isolation of gonadal tissues. However, contamination with other cell types is still possible. Another problem is that possible sex-related effects cannot be known. In addition, the general lower level of gene expression early in development may hamper finding differences regardless whether the animals have been treated or not with chemicals to decipher some physiological regulation. In contrast, RNA harvested after both the process being studied and the treatment applied to modify are completed has advantage in that, because the gonads have had time to develop, they are easier to dissect out, such that the effects can be studied separately for each sex and that problems of low gene expression are no longer found. As a major drawback, this second approach has disadvantage in that measured changes may be the consequence rather than the cause. Another potential problem is the risk of loosing the capability of measuring effects if those happen to be reversible.

### GSD Species with Monosex Populations Available

In species with a monofactorial system of sex determination such as XX/XY or ZW/ZZ, the production of monosex stocks is feasible. One such species is the rainbow trout, where a great deal of work has been carried out to study genes mainly implicated in sex differentiation. Transcriptomic studies of a hundred of candidate genes responsible for gonad differentiation in all-female populations of rainbow trout revealed that masculinization by androgen (11ß-hydroxyandrostenedione) treatment involved a de-differentiating process in the granulosa cells of differentiating females (Baron et al. 2007, 2008). The hormonal masculinization with androgen first repressed genes related to granulosa cells involved in early ovarian differentiation (e.g., *foxl2*, *cyp19a1*) and then decreased other key genes involved in the early oogenesis process (e.g., *gdf9*, *fshb*, *lhb*). In addition, some Sertoli cell markers were upregulated (e.g., *sox9*, *dmrt1*), although some Leydig cell markers were downregulated (e.g., *star*, *cyp11*). Interestingly, when masculinization was achieved by inhibition of *cyp19a1*, Sertoli and Leydig cell markers were restored, indicating that masculinization by inhibition of estrogen synthesis resembles more the natural physiological process of masculinization than that achieved by direct androgen treatment (Vizziano et al. 2008). In contrast, when feminization was induced by estrogen treatment, it was found that, as expected, many genes related with early ovarian differentiation, particularly *foxl2*, were upregulated. However, estrogen treatment did not repress genes involved in testis differentiation such as *dmrt1* or *sox9a1*, indicating that what is essential for female differentiation is the upregulation of a set of ovarian-specific genes (Vizziano-Cantonnet et al. 2008).

Also in rainbow trout, Cavileer et al. (2009) used a custom microarray and a strategy based on identifying strong associations in gene expression patterns between known sex differentiation genes (target genes) and novel genes (target-associated genes) previously not allied with sex differentiation in fishes. With this approach, several novel genes were identified in the gonads of embryonic female and male rainbow trout that could be involved in sex-specific differentiation pathways in this fish (Cavileer et al. 2009).

In Nile tilapia, another species where monosex populations can be created, the gonadal expression of *foxl2* and *cyp19a1* in XX gonads and *dmrt1* in XY gonads at early stages of development (5–6 days after hatch), was found to be critical for undifferentiated gonads to differentiate into ovary or testis, respectively (Ijiri et al. 2008). Other steroidogenic enzymes necessary for the synthesis of estrogens were detected during the early stages of gonad differentiation, suggesting that estradiol-17β plays a critical role in ovarian differentiation in tilapia. Recently, functional molecular in vitro and in vivo studies in this fish species revealed that *dmrt1* could directly bind to the aromatase promoter. The transgenic fish overexpressing *dmrt1* had decreased aromatase transcription levels in gonads and consequently lower 17β-estradiol plasma levels (Wang et al. 2010). Also in tilapia, transcriptomic analysis in the gonads of monosex fish revealed a total of 22,385 expressed sequence tags (ESTs) in the ovary and testis (Wang 2011). Furthermore, approximately 70,000 sequences from gonadal samples were identified from 3-month-old Nile tilapia, with more genes being expressed at comparatively higher levels in the testis than in the ovary (Huang et al. 2011). The latter study is interesting towards our understanding of regulation of gametogenesis but less useful to understand sex differentiation because 3-month-old Nile tilapia are already sexually differentiated and are engaged in active gametogenesis.

An aspect that has not received the attention it deserves is the genetic background of the fish used in these types of experiments. Ideally, the genetic variation present should be representative of the species or the strain used, and it should be similar between all-female and all-male populations so differences in gene expression are only due to sex. This issue is particularly relevant when gynogenetic fish are used to create all-female stocks since gynogenetic diploid fish, especially if they are mitogynogens, have a much higher level of homozygosis than normal diploids. Thus, gene expression data obtained from all-female gynogenetic diploids should always be interpreted with caution considering the possible influence of reduced genetic variation.

### GSD Species Without Monosex Populations Available

The zebrafish (*Danio rerio*) has become an important vertebrate model for basic and biomedical research. This model is also contributing to decipher various aspects of fish reproductive biology, especially when sex differentiation is analyzed at the whole gonad transcriptome level (Li et al. 2004). However, the processes of sex determination and sex differentiation in zebrafish are still not well understood. During the first stages of gonad development, primordial germ cells (PGCs) migrate to the genital ridge and their presence is required for the formation of the female gonads. If their presence is inhibited in various ways, the absence of PGCs will lead to testis differentiation (Siegfried and Nusslein-Volhard 2008; Siegfried 2010). At around 24 days post-fertilization (dpf), juvenile ovaries either enter an apoptotic pathway which stops ovarian development and testis development follows or continue with ovarian differentiation depending upon the genetic constitution (Orban et al. 2009). Studies carried out with steroid-treated populations of zebrafish have led to the hypothesis that testis and ovarian differentiation is supported by an underlying ZZ/ZW chromosomal system (Tong et al. 2010). However, steroid-treated populations should be used with caution since results may not reflect the normal process of sex differentiation. Furthermore, the environmental component regulating sex determination—still poorly defined nowadays in this species—needs to be also taken into consideration (Ospina-Álvarez and Piferrer 2008; Orban et al. 2009). Analysis of the transcriptome of the zebrafish ovary and testis during gonad differentiation was achieved by using a specific microarray with over 47,000 ESTs (Li et al. 2004). Later, other transcriptomic analysis provided 116,638 gonad-derived zebrafish ESTs and revealed novel genes with sexually dimorphic expression (Sreenivasan et al. 2008), also showing a higher abundance of transcripts in male than in female gonads. Males also had many more expression differences between body and gonads than females did (Small et al. 2009). Recent proteomic studies provided the most comprehensive list of proteins expressed in mature zebrafish gonads, establishing the basis to elucidate processes occurring during fish gonad development from a protein-based perspective (Groh et al. 2011).

In species with environmental influences on sex determination and differentiation, experiments to study gene expression patterns during sex differentiation should ideally be carried out under different levels of the main environmental variable, e.g., temperature in species with TSD (Fig. 4c). This, however, is not always feasible and increases experimental costs significantly. In the pejerrey, utilization of a medaka microarray has enabled to identify putative genes involved in sex differentiation in animals reared at male- and female-producing temperatures (Fernandino et al. 2011).

## Environmental Effects on Gene Expression

**Fig. 6:**
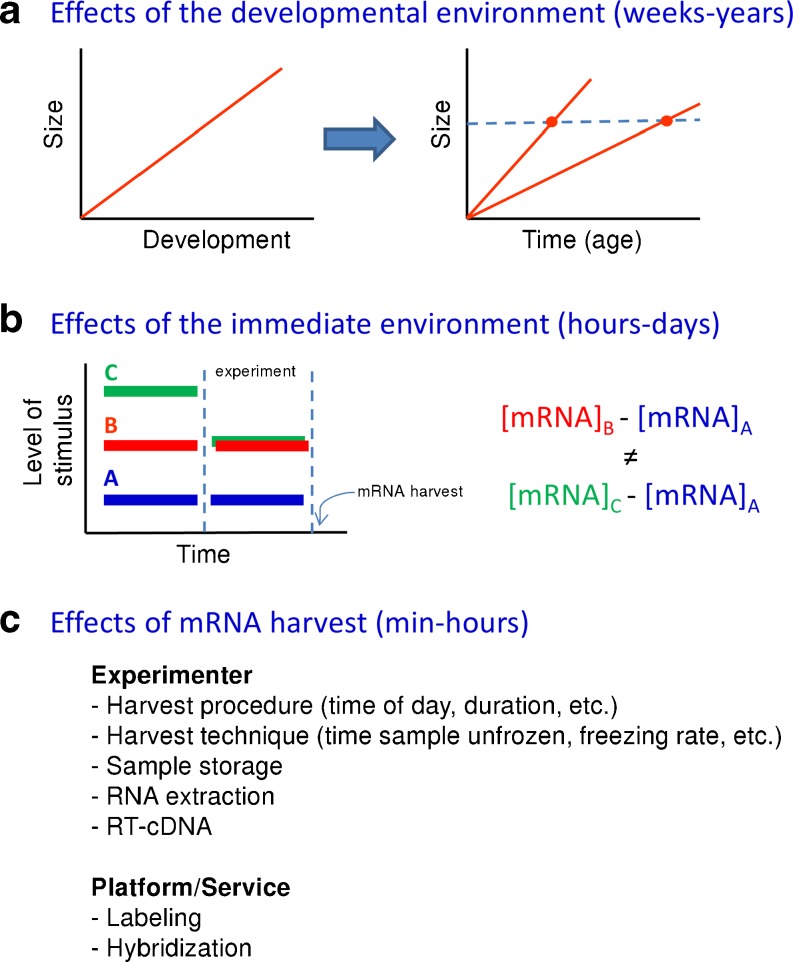
Sources of environmental influences on gene expression levels. **a** Effects of the developmental environment, typically in the range of weeks–years. Size and development are positively correlated. If size is used as a proxy for development, it may give confounding effects if two individuals with the same size but with different developmental rates are compared. **b** Effects of the immediate environment, usually in the range of hours–days. An experiment is carried out (delimited by the *two vertical dashed lines* in time) and RNA is harvested at the end. Comparing mRNA levels of a population with a certain level of stimulus (*red* or *green bar* with the duration of the experiment) against a control population (*blue*) will give different results depending on the immediate environment prior the start of the experiment. **c** Effects of RNA harvest (in the range of minutes–hours). Factors that affect mRNA levels are listed

From a pure experimental point of view, there are several potential sources of environmental influences that need to be under control in order to avoid confounding results when studying gene expression levels (Hodgins-Davis and Townsend 2009) (Fig. 6). When these considerations are applied to the study of gene expression during sex determination and differentiation in fish, we have found that one of these sources is constituted by the effects of the developmental environment, typically in the range of weeks–years. Size is positively correlated with development in fish. However, using size as a proxy for development may give confounding effects if two individuals attain the same size at different ages, a consequence of different developmental rates (Fig. 6a). Effects of the rearing environment are also possible in the sense that, many times, monosex male and female populations or treated vs. untreated fish are kept in separate tanks, although it might be expected that differential expression of major genes involved in sex differentiation is maintained throughout life regardless of the rearing environment. Rearing the monosex population in a common garden setting is possible (and advisable) if genotyping is feasible but rearing hormonally treated and untreated fish in the same tank is difficult or impossible. Another potential source of confounding effects is the so-called immediate environment, usually in the range of hours–days. Comparing mRNA levels of a population with a certain level of stimulus against a control population will likely give different results depending on the immediate environment that the animals have been kept in prior the start of the experiment (Fig. 6b). Finally, another source of variation is the effect of RNA harvest, usually in the range of minutes–hours. Many factors affect mRNA levels; some of them need to be taken into account by the experimenter, while others usually are dealt with by the platform or service (e.g., microarray platform) (Fig. 6c).

**Fig. 7:**
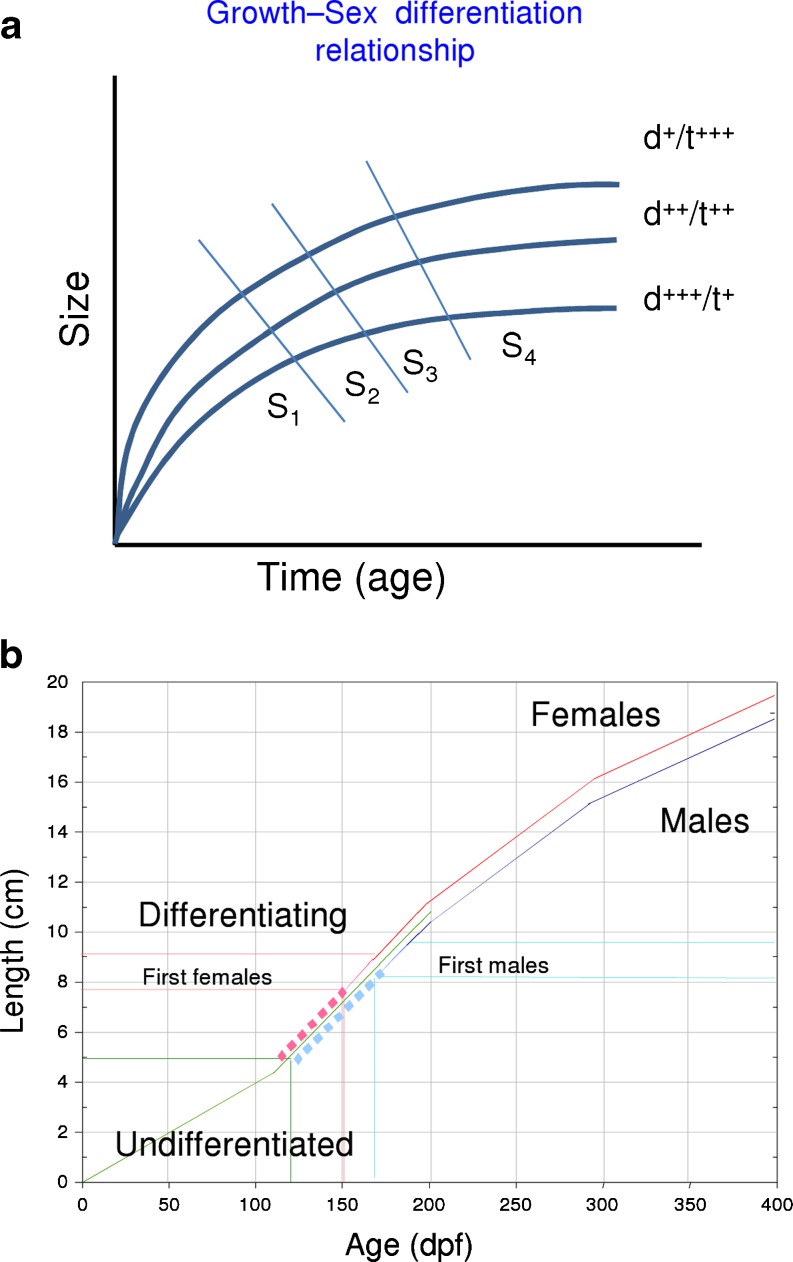
Effects of developmental time or age (in dpf) on fish size and the process of sex differentiation. **a** Different levels (*+*, *++*, *+++*) of environmental factors such as rearing density (*d*) or temperature (*t*), with opposing effects, alter the age–size relationship and, in turn, the time at which a given stage (*S*) of development are first attained. **b** Age–length relationship in European sea bass and sex differentiation. Indicated are the different phenotypes

On the other hand, there are sources of variation directly linked with the particular biology of the species considered. One clear example of genotype × environment interaction concerns European sea bass (Fig. 7), where sex ratios are the product of both genetic (polygenic sex determination mechanism) (Vandeputte et al. 2007) and environmental (temperature) influences (Piferrer et al. 2005). In this species, several studies have shown that there is an early association between growth rates and sex (Blázquez et al. 1999), with females being among the largest fish and males being among the smallest ones (Blázquez et al. 1999; Saillant et al. 2001; Vandeputte et al. 2007). In addition, sexual growth dimorphism is present at the time of marketing when animals reach 300–400 g (Saillant et al. 2001; Navarro-Martín et al. 2009a). Repetitive size grading of a European sea bass population at early stages generates one female-dominant population with the largest fish and one male-dominant population with the smallest ones (Papadaki et al. 2005). Female-dominant populations were also obtained after a single size grading (Koumoundouros et al. 2002 and Saillant et al. 2003), suggesting an early association between growth rates and sex phenotype.

Studies in mammals have revealed the importance of a threshold in gonad development that needs to be reached before a specific time. Whether this threshold is reached or not influences gonadal fate (Mittwoch 1989). A similar principle has been suggested for fishes (“Kraak and De Looze, 1993). Thus, sex would be depending on growth. In fish, in general, studies with different species such as eel (*Anguilla anguilla*) (Colombo et al. 1984; Roncarati et al. 1997), roach (*Rutilus rutilus*) (Paull et al. 2009), and zebrafish (Lawrence et al. 2008) evidence the relationship between early high growth and female differentiation. Interestingly, two significant QTLs, one affecting both body weight and sex and another affecting sex, were detected on the same linkage group in the protandrous hermaphrodite sea bream, *Sparus aurata* (Loukovitis et al. 2011), also suggesting this relationship between growth and sex in hermaphrodites. However, studies with steroid-treated European sea bass showed no differences in growth neither between normal females and estradiol-17ß feminized females (Saillant et al. 2001) nor between normal males and fadrozole (an aromatase inhibitor)-masculinized males (Navarro-Martín et al. 2009b), suggesting that growth depends on phenotypic sex.

Recent experiments with European sea bass have shown that reduced growth with or without previous size grading of the population during the sex differentiation period (8–12 cm standard length) did not affect sexual differentiation. Rather, the final sex ratios were related to growth rates at the time of the size grading, indicating that the association between sex and growth rates is established earlier than previously thought (N. Díaz, L. Ribas and F. Piferrer, unpublished observations). Furthermore, this was independent of the occurrence of compensatory growth, a phenomenon whereby growth rates increase and size converges between fish previously subjected to different growth rates (Ali et al. 2003; Jobling 2010). To further explore this relationship, a European sea bass microarray has been used to compare gene expression in the differentiating testis of males from the fast- vs. the slow-growing groups. Preliminary results indicate differences in the expression of important genes related to reproduction such as the anti-Müllerian hormone, *dmrt1*, or the steroidogenic enzyme aromatase (*cyp19a1*). Together these results illustrate not only that the final sex ratio of the population is established well before the first signs of sex differentiation are visible but also that it is not affected by growth rates during but before sex differentiation. It should be noted that in experiments on species where environmental factors influence sex ratios, caution in the experimental design should be taken. Thus, when looking for genotype × environment effects, it is usually advisable to reduce the number of genetic (families, strains …) and environmental conditions and to maintain the highest possible number of individuals rather than to increase the number of combinations at the expense of reducing sample sizes.

## Contribution of Epigenetics

Epigenetics is a very active area of research nowadays. A major epigenetic mechanism involves changes in the methylation of DNA. When these changes occur in CpG nucleotides located within the promoter region of genes, they significantly contribute to changes in gene expression. Epigenetic mechanisms involving DNA methylation have been reported during the normal development of zebrafish (Mhanni and McGowan 2004), are implicated in the activation and silencing of the sex-determining gene, SrY, in mammals (Nishino et al. 2004), and in the responses to temperature in plants (Sung and Amasino 2004). Epigenetic mechanisms have also been implicated in the reproductive alterations following exposure to pollutants (Zama and Uzumcu 2010).

**Fig. 8:**
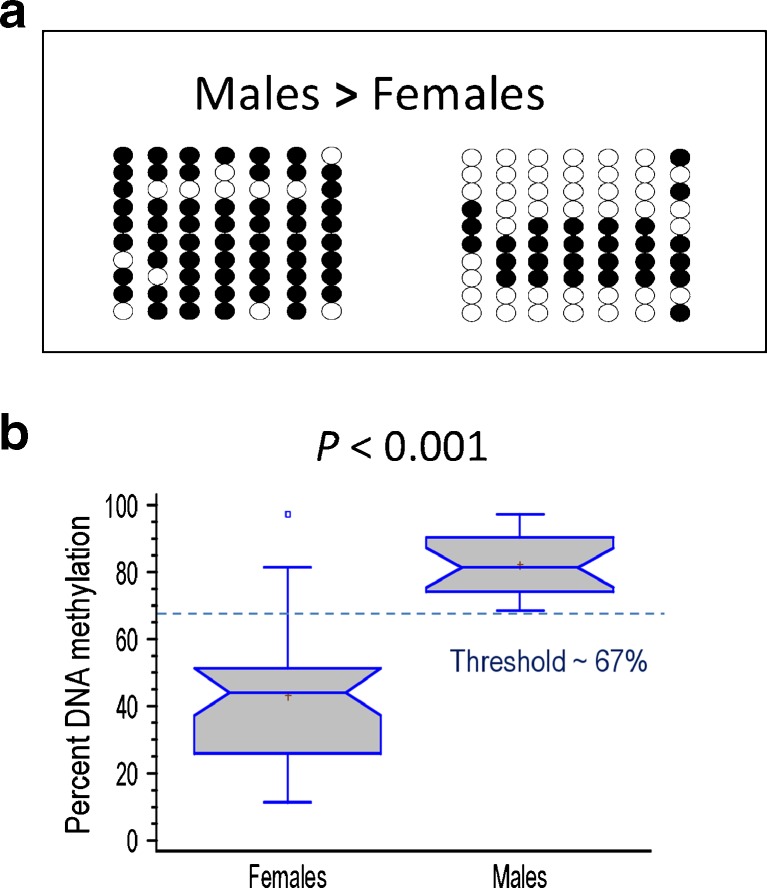
Potential contribution of epigenetics in understanding changes in gene expression in fish. Illustrated are the differences in DNA methylation of the gonadal aromatase (*cyp19a1*) promoter of European sea bass between males and females. This promoter contains seven CpGs (*columns*) within the first 600 bp. **a** Ten clones (*rows*) of a typical male and a typical female are represented, showing unmethylated (*open circle*) or methylated (*filled circle*) CpGs. **b** *Box plot* of actual DNA methylation levels between sexes. Data redrawn from Navarro-Martín et al. (2011)

It has been argued that embryonic and larval fishes are particularly well suited for epigenetic research in a variety of aspects including, but not limited to, feeding behavior, food conversion, and hypoxic tolerance, as reviewed by Burggren and Blank (2009). Technical advantages of fish include external fertilization, relatively high fecundity, and short life cycle. An example of an epigenetic effect is the observation that adult zebrafish chronically exposed to 2–4 weeks of moderate hypoxia and then returned to normoxia subsequently produced offspring that exhibited enhanced hypoxic resistance. Thus, it is not difficult to ascertain that these aspects are of extraordinary importance in the context of modern, intensive fish farming, where conditions may be suboptimal due to high rearing densities. Zebrafish and European sea bass are two of the richest species in terms of genomic resources (Kuhl et al. 2010) where to begin to integrate epigenetics into aquaculture research.

Gorelick (2003) proposed that in animals with TSD sex differences were first determined by different patterns in DNA methylation and also that environmental variations may change those patterns. Earlier studies in our laboratory showed that *cyp19a1* not only was an early marker of ovarian differentiation but also that the expression of this gene was inhibited by high temperature (Blázquez et al. 2009). However, the mechanism connecting external temperature and aromatase, although essential for sex differentiation in species with TSD, including reptiles and fish, has remained elusive (Lance 2009; Luckenbach et al., 2009).

In European sea bass, the masculinizing effects of high temperature are maximal during early development (Navarro-Martín et al. 2009a) not only well before the sexual differentiation of the gonads but even before the formation of the gonadal ridges themselves. This suggests that an epigenetic mechanism may be involved in transducing environmental temperature in changes in gene expression. Thus, it was conceivable that an epigenetic mechanism could be part of the sex ratio response to temperature, a question never before explored. Research on this subject in our laboratory with 1-year-old European sea bass showed that the *cyp19a1* promoter was hypermethylated in males when compared to females (Navarro-Martín et al. 2011), in agreement with the well-established lower levels of *cyp19a1* expression observed in testes than in ovaries (Fig. 8). Furthermore, we also found that high temperature increased the methylation levels of *cyp19a1* in both sexes. Finally, luciferase assays confirmed that methylation of the promoter causes the in vitro repression of transcription, suggesting that *cyp19a1* promoter methylation is the mechanism by which *cyp19a1* transcription is silenced in developing males during sex differentiation, preventing the differentiation of the undifferentiated gonad into an ovary (Navarro-Martín et al. 2011).

The analysis of epigenetic changes in response to varying environments will be very relevant in the coming years. The analysis at the whole genome (epigenome) level still poses important challenges (Laird 2010), but it will benefit from NGS technologies. It is expected that only at this level will the most significant contributions brought by integrating epigenetics be made.

## Concluding Remarks and Future Prospects

Fish sex ratios are the result of a complex combination of genetic, biochemical, and environmental interactions. The ultimate result of these interactions at the individual levels is gender, male or female. However, at the population level, the combination of sex determination and differentiation sets the sex ratio. In turn, sex ratios define the reproductive capacity of populations and, if sex growth dimorphism exists, also the growth characteristics, something very important in an aquaculture context. Furthermore, despite some efforts having been put into this direction, the relationship between fitness, sex, and growth deserves further research. To this end, NGS techniques can help to find sex-determining genes and molecular markers of sex differentiation in fish, by far not only the most diverse group of vertebrates in terms of number of species but also, specially, in terms of reproductive strategies and sex-determining mechanisms. The sequencing of the genome in new species will eventually help to understand the great diversity and evolution of sex-determining mechanisms in this vast group of animals characterized by rapid transitions between alternate mechanisms. In addition, more sequencing and functional genomics data will contribute to better annotation of the probes already available in microarrays.

Proteomics, analysis of gene networks and studies of non-coding RNAs, are still limited in fish genomics in general and in the context of sex determination/differentiation in particular; so, implementation of this type of approaches will likely bear fruitful results.

In many species of fish, the existence of strong G × E interactions may lead to complex reaction norms connecting the variation of a given environmental factor and phenotype (sex ratios). In this context, it is envisaged that epigenetic studies will become more common and will help to understand how the environment controls gene expression related to production traits.

Finally, the development of molecular probes to identify sex or the establishment of molecular signatures characteristic of a given phenotype can have an immediate application to the fish farming industry for broodstock selection and monosex production for better performance.

## References

[CR1] Ali M, Nicieza A, Wootton RJ (2003). Compensatory growth in fishes: a response to growth depression. Fish Fish.

[CR2] Aranda G, Aragón L, Corriero A, Mylonas CC, de la Gándara F, Belmonte A, Medina, A. (2011) GnRHa-induced spawning in cage-reared Atlantic bluefin tuna, An evaluation using stereological quantification of ovarian post-ovulatory follicles. Aquaculture 317:255–259

[CR3] Baron D, Montfort J, Houlgatte R, Fostier A, Guiguen Y (2007). Androgen-induced masculinization in rainbow trout results in a marked dysregulation of early gonadal gene expression profiles. BMC Genom.

[CR4] Baron D, Houlgatte R, Fostier A, Guiguen Y (2008). Expression profiling of candidate genes during ovary-to-testis trans-differentiation in rainbow trout masculinized by androgens. Gen Comp Endocrinol.

[CR5] Blázquez M, Carrillo M, Zanuy S, Piferrer F (1999). Sex ratios in offspring of sex-reversed sea bass and the relationship between growth and phenotypic sex differentiation. J Fish Biol.

[CR6] Blázquez M, Navarro-Martin L, Piferrer F (2009). Expression profiles of sex differentiation-related genes during ontogenesis in the European sea bass acclimated to two different temperatures. J Exp Zool B Mol Dev Evol.

[CR7] Breder CM, Rosen DE (1966). Modes of reproduction in fishes.

[CR8] Burggren W, Blank T (2009). Physiological study of larval fishes: challenges and opportunities. Sci Mar.

[CR9] Cavileer T, Hunter S, Okutsu T, Yoshizaki G, Nagler JJ (2009). Identification of novel genes associated with molecular sex differentiation in the embryonic gonads of rainbow trout (*Oncorhynchus mykiss*). Sex Dev.

[CR10] Cerdà J, Mercadé J, Lozano JJ, Manchado M, Tingaud-Sequeira A, Astola A, Infante C, Halm S, Viñas J, Castellana B, Asensio E, Cañavate P, Martínez-Rodríguez G, Piferrer F, Planas JV, Prat F, Yúfera M, Durany O, Subirada F, Rosell E, Maes T (2008). Genomic resources for a commercial flatfish, the Senegalese sole (*Solea senegalensis*): EST sequencing, oligo microarray design, and development of the Soleamold bioinformatic platform. BMC Genomics.

[CR11] Colombo G, Grandi G, Rossi R (1984). Gonad differentiation and body growth in *Anguilla anguilla* L. J Fish Biol.

[CR12] Ezaz MT, Harvey SC, Boonphakdee C, Teale AJ, McAndrew BJ, Penman DJ (2004). Isolation and physical mapping of sex-linked AFLP markers in Nile tilapia, *Oreochromis niloticus* L. Mar Biotechnol.

[CR13] Felip A, Young WP, Wheeler PA, Thorgaard GH (2005). An AFLP-based approach for the identification of sex-linked markers in rainbow trout, *Oncorhynchus mykiss*. Aquaculture.

[CR14] Felip A, Zanuy S, Carrillo M (2006). Comparative analysis of growth performance and sperm motility between precocious and non-precocious males in the European sea bass (*Dicentrarchus labrax*, L.). Aquaculture.

[CR15] Fernandino JL, Popesku JT, Paul-Prasanth B, Xiong H, Hattori RS, Oura M, Strüssmann CA, Somoza GM, Matsuda M, Nagahama Y, Trudeau VL (2011). Analysis of sexually dimorphic expression of genes at early gonadogenesis of pejerrey *Odontesthes bonariensis* using a heterologous microarray. Sex Dev.

[CR16] Ferraresso S, Milan M, Pellizzari C, Vitulo N, Reinhardt R, Canario AV, Patarnello T, Bargelloni L (2010). Development of an oligo DNA microarray for the European sea bass and its application to expression profiling of jaw deformity. BMC Genomics.

[CR17] Fraser BA, Weadick CJ, Janowitz I, Rodd FH, Hughes KA (2011). Sequencing and characterization of the guppy (*Poecilia reticulata*) transcriptome. BMC Genomics.

[CR18] Garcia-Reyero N, Griffitt RJ, Liu L, Kroll KJ, Farmerie WG, Barber DS, Denslow ND (2008). Construction of a robust microarray from a non-model species largemouth bass, *Micropterus salmoides* (Lacepede), using pyrosequencing technology. J Fish Biol.

[CR19] Gorelick R (2003). Evolution of dioecy and sex chromosomes via methylation driving Muller's ratchet. Biol J Linn Soc.

[CR20] Groh KJ, Nesatyy VJ, Segner H, Eggen RIL, Suter MJF (2011). Global proteomics analysis of testis and ovary in adult zebrafish (*Danio rerio*). Fish Physiol Biochem.

[CR21] Grossen C, Neuenschwander S, Perrin N (2010). Temperature-dependent turnovers in sex-determination mechanisms: a quantitative model. Evolution.

[CR22] Guiguen Y, Fostier A, Piferrer F, Chang CF (2010). Ovarian aromatase and estrogens: a pivotal role for gonadal sex differentiation and sex change in fish. Gen Comp Endocrinol.

[CR23] Haffray P, Lebegue E, Jeu S, Guennoc M, Guiguen Y, Baroiller JF, Fostier A (2009). Genetic determination and temperature effects on turbot *Scophthalmus maximus* sex differentiation: an investigation using steroid sex-inverted males and females. Aquaculture.

[CR24] Hale MC, McCormick CR, Jackson JR, DeWoody JA (2009). Next-generation pyrosequencing of gonad transcriptomes in the polyploid lake sturgeon (*Acipenser fulvescens*): the relative merits of normalization and rarefaction in gene discovery. BMC Genomics.

[CR25] Hale MC, Jackson JR, DeWoody JA (2010). Discovery and evaluation of candidate sex-determining genes and xenobiotics in the gonads of lake sturgeon (*Acipenser fulvescens*). Genetica.

[CR26] Herpin A, Schartl M (2011). Dmrt1 genes at the crossroads: a widespread and central class of sexual development factors in fish. FEBS J.

[CR27] Hodgins-Davis A, Townsend JP (2009). Evolving gene expression: from G to E to G × E. Trends Ecol Evol.

[CR28] Huang BF, Sun YL, Wang DS (2011) Transcriptome analysis of the artificially induced sex reversal in the Nile tilapia. 9th Int Symp Reprod Physiol Fish, Cochin, India (abstract)

[CR29] Ijiri S, Kaneko H, Kobayashi T, Wang DS, Sakai F, Paul-Prasanth B, Nakamura M, Nagahama Y (2008). Sexual dimorphic expression of genes in gonads during early differentiation of a teleost fish, the Nile tilapia *Oreochromis niloticus*. Biol Reprod.

[CR30] Imsland AK, Folkvord A, Grung GL, Stefansson SO, Taranger GL (1997). Sexual dimorphism in growth and maturation of turbot, *Scophthalmus maximus* (Rafinesque, 1810). Aquac Res.

[CR31] Jobling M (2010). Are compensatory growth and catch-up growth two sides of the same coin?. Aquacult Int.

[CR32] Karube M, Fernandino JI, Strobl-Mazzulla P, Strüssmann CA, Yoshizaki G, Somoza GM, Patiño R (2007). Characterization and expression profile of the ovarian cytochrome p-450 aromatase (*cyp19a1*1) gene during thermolabile sex determination in pejerrey, *Odontesthes bonariensis*. J Exp Zool Part A.

[CR33] Kitano T, Takamune K, Kobayashi T, Nagahama Y, Abe SI (1999). Suppression of P450 aromatase gene expression in sex-reversed males produced by rearing genetically female larvae at a high water temperature during a period of sex differentiation in the Japanese flounder (*Paralichthys olivaceus*). J Mol Endocrinol.

[CR34] Koumoundouros G, Pavlidis M, Anezaki L, Kokkari C, Sterioti K, Divanach P, Kentouri M (2002). Temperature sex determination in the European sea bass, *Dicentrarchus labrax* (L., 1758) (Teleostei, Perciformes, Moronidae): critical sensitive ontogenetic phase. J Exp Zool.

[CR35] Kovacs B, Egedi S, Bartfai R, Orban L (2000). Male-specific DNA markers from African catfish, *Clarias gariepinus*. Genetica.

[CR36] Kraak SBM, Delooze EMA (1993). A new hypothesis on the evolution of sex determination in vertebrates—big females ZW, big males XY. Neth J Zool.

[CR37] Kuhl H, Beck A, Wozniak G, Canario A, Volckaert F, Reinhardt R (2010). The European sea bass *Dicentrarchus labrax* genome puzzle: comparative BAC-mapping and low coverage shotgun sequencing. BMC Genomics.

[CR38] Laird PW (2010). Principles and challenges of genome-wide DNA methylation analysis. Nat Rev Gen.

[CR39] Lance V (2009) Is regulation of aromatase expression in reptiles the key to understanding temperature-dependent sex determination? J Exp Zool 311A:314–322 10.1002/jez.465 18668631

[CR40] Lawrence C, Ebersole JP, Kesseli RV (2008). Rapid growth and out-crossing promote female development in zebrafish (*Danio rerio*). Environ Biol Fish.

[CR41] Li Y, Chia JM, Bartfai R, Christoffels A, Yue GH, Ding K, Ho MY, Hill JA, Stupka E, Orban L (2004). Comparative analysis of the testis and ovary transcriptomes in zebrafish by combining experimental and computational tools. Comp Funct Genom.

[CR42] Loukovitis D, Sarropoulou E, Tsigenopoulos CS, Batargias C, Magoulas A, Apostolidis AP, Chatziplis D, Kotoulas G (2011). Quantitative trait loci involved in sex determination and body growth in the gilthead sea bream (*Sparus aurata* L.) through targeted genome scan. PLoS One.

[CR43] Luckenbach JA, Borski RJ, Daniels HV, Godwin J (2009). Sex determination in flatfishes: mechanisms and environmental influences. Sem Cell Dev Biol.

[CR44] Magerhans A, Hörstgen-Schwark G (2010). Selection experiments to alter the sex ratio in rainbow trout (*Oncorhynchus mykiss*) by means of temperature treatment. Aquaculture.

[CR45] Martínez P, Bouza C, Hermida M, Fernández J, Toro MA, Vera M, Pardo B, Millán A, Fernández C, Vilas R, Viñas A, Sánchez L, Felip A, Piferrer F, Ferreiro I, Cabaleiro S (2009). Identification of the major sex-determining region of turbot (*Scophthalmus maximus*). Genetics.

[CR46] Matsuda M, Nagahama Y, Shinomiya A, Sato T, Matsuda C, Kobayashi T, Morrey CE, Shibata N, Asakawa S, Shimizu N, Hori H, Hamaguchi S, Sakaizumi M (2002). DMY is a Y-specific DM-domain gene required for male development in the medaka fish. Nature.

[CR47] Mhanni AA, McGowan RA (2004). Global changes in genomic methylation levels during early development of the zebrafish embryo. Dev Genes Evol.

[CR48] Mittwoch U (1989). Sex-differentiation in mammals and tempo of growth—probabilities vs switches. J Theor Biol.

[CR49] Munger SC, Aylor DL, Syed HA, Magwene PM, Threadgill DW, Blanche C (2009). Elucidation of the transcription network governing mammalian sex determination by exploiting strain-specific susceptibility to sex reversal. Genes Devel.

[CR50] Nanda I, Kondo M, Hornung U, Asakawa S, Winkler C, Shimizu A, Shan Z, Haaf T, Shimizu N, Shima A, Schmid M, Schartl M (2002). A duplicated copy of DMRT1 in the sex-determining region of the Y chromosome of the medaka, *Oryzias latipes*. PNAS.

[CR51] Navarro-Martín L, Blázquez M, Vinas J, Joly S, Piferrer F (2009). Balancing the effects of rearing at low temperature during early development on sex ratios, growth and maturation in the European sea bass (*Dicentrarchus labrax*). Limitations and opportunities for the production of highly female-biased stocks. Aquaculture.

[CR52] Navarro-Martín L, Blázquez M, Piferrer F (2009). Masculinization of the European sea bass (*Dicentrarchus labrax)* by treatment with an androgen or aromatase inhibitor involves different gene expression and has distinct lasting effects on maturation. Gen Comp Endocrinol.

[CR53] Navarro-Martín L, Viñas J, Ribas L, Díaz N, Gutiérrez A, Di Croce L, Piferrer F (2011) DNA methylation of the gonadal aromatase (*cyp19a*) promoter is involved in temperature-dependent sex ratio shifts in the European sea bass. PLoS Genetics 7(12): e1002447 10.1371/journal.pgen.1002447 PMC3248465 22242011

[CR54] Nishino K, Hattori N, Tanaka S, Shiota K (2004). DNA methylation-mediated control of Sry gene expression in mouse gonadal development. J Biol Chem.

[CR55] Orban L, Sreenivasan R, Olsson PE (2009). Long and winding roads: testis differentiation in zebrafish. Mol Cell Endocrinol.

[CR56] Ospina-Alvarez N, Piferrer F (2008). Temperature-dependent sex determination in fish revisited: prevalence, a single sex ratio response pattern, and possible effects of climate change. PLoS One.

[CR57] Papadaki M, Piferrer F, Zanuy S, Maingot E, Divanach P, Mylonas CC (2005). Growth, sex differentiation and gonad and plasma levels of sex steroids in male- and female-dominant populations of *Dicentrarchus labrax* obtained through repeated size grading. J Fish Biol.

[CR58] Parker GA (1992). The evolution of sexual size dimorphism in fish. J Fish Biol.

[CR59] Paull G, Filby A, Tyler C (2009). Growth rate during early life affects sexual differentiation in roach (*Rutilus rutilus*). Environ Biol Fish.

[CR60] Penman DJ, Piferrer F (2008). Fish gonadogenesis. Part I: genetic and environmental mechanisms of sex determination. Rev Fish Sci.

[CR61] Piferrer F (2001). Endocrine sex control strategies for the feminization of teleost fish. Aquaculture.

[CR62] Piferrer F, Farrell AP (2011). Endocrine control of sex differentiation in fish. Encyclopedia of fish physiology, from gene to environment.

[CR63] Piferrer F, Guiguen Y (2008). Fish gonadogenesis. Part II: molecular biology and genomics of sex differentiation. Rev Fish Sci.

[CR64] Piferrer F, Blázquez M, Navarro L, Gonzalez A (2005). Genetic, endocrine, and environmental components of sex determination and differentiation in the European sea bass (*Dicentrarchus labrax* L.). Gen Comp Endocrinol.

[CR65] Piferrer F, Beaumont A, Falguière JC, Flajshans M, Haffray P, Colombo L (2009). The use of induced polyploidy in the aquaculture of bivalves and fish for performance improvement and genetic containment. Aquaculture.

[CR66] Qvarnström A, Bailey RI (2009). Speciation through evolution of sex-linked genes. Heredity.

[CR67] Roncarati A, Mordenti O, Acciarri S, Quagliarini C, Benedetti S, Angellotti L, Melotti P (1997). Use of different feeding regimes in the larval rearing of striped bass (*Morone saxatilis* Walbaum). Riv Ital Acquacoltura.

[CR68] Saillant E, Fostier A, Menu B, Haffray P, Chatain B (2001). Sexual growth dimorphism in sea bass *Dicentrarchus labrax*. Aquaculture.

[CR69] Saillant E, Chatain B, Menu B, Fauvel C, Vidal M, Fostier A (2003). Sexual differentiation and juvenile intersexuality in the European sea bass (*Dicentrarchus labrax*). J Zool.

[CR70] Sarropoulou E, Nousdili D, Magoulas A, Kotoulas G (2008). Linking the genomes of nonmodel teleosts through comparative genomics. Mar Biotechnol.

[CR71] Shao CW, Chen SL, Scheuring CF, Xu JY, Sha ZX, Dong XL, Zhang HB (2010). Construction of two BAC libraries from half-smooth tongue sole *Cynoglossus semilaevis* and identification of clones containing candidate sex-determination genes. Mar Biotechnol.

[CR72] Shapiro MD, Summers BR, Balabhadra S, Aldenhoven JT, Miller AL, Cunningham CB, Bell MA, Kingsley DM (2009). The genetic architecture of skeletal convergence and sex determination in ninespine sticklebacks. Curr Biol.

[CR73] Siegfried KR (2010). In search of determinants: gene expression during gonadal sex differentiation. J Fish Biol.

[CR74] Siegfried KR, Nusslein-Volhard C (2008). Germ line control of female sex determination in zebrafish. Dev Biol.

[CR75] Small CM, Carney GE, Mo QX, Vannucci M, Jones AG (2009). A microarray analysis of sex- and gonad-biased gene expression in the zebrafish: evidence for masculinization of the transcriptome. BMC Genomics.

[CR76] Sreenivasan R, Cai MN, Bartfai R, Wang XG, Christoffels A, Orban L (2008). Transcriptomic analyses reveal novel genes with sexually dimorphic expression in the zebrafish gonad and brain. PLoS One.

[CR77] Sung SB, Amasino RM (2004). Vernalization in *Arabidopsis thaliana* is mediated by the PHD finger protein VIN3. Nature.

[CR78] Tanaka K, Takehana Y, Naruse K, Hamaguchi S, Sakaizumi M (2007). Evidence for different origins of sex chromosomes in closely related *Oryzias* fishes: substitution of the master sex-determining gene. Genetics.

[CR79] Tingaud-Sequeira A, Chauvigne F, Lozano J, Agulleiro MJ, Asensio E, Cerda J (2009). New insights into molecular pathways associated with flatfish ovarian development and atresia revealed by transcriptional analysis. BMC Genomics.

[CR80] Tong SK, Hsu HJ, Chung BC (2010). Zebrafish monosex population reveals female dominance in sex determination and earliest events of gonad differentiation. Develop Biol.

[CR81] Uller T, Helanterä H (2011). From the origin of sex determining factors to the evolution of sex-determining systems. Quart Rev Biol.

[CR82] van Nes S, Andersen O (2006). Temperature effects on sex determination and ontogenetic gene expression of the aromatases *cyp19a1* and cyp19b, and the estrogen receptors *esr1* and *esr2* in Atlantic halibut (*Hippoglossus hippoglossus*). Mol Reprod Dev.

[CR83] Vandeputte M, Dupont-Nivet M, Chavanne H, Chatain B (2007). A polygenic hypothesis for sex determination in the European sea bass *Dicentrarchus labrax*. Genetics.

[CR84] Vizziano D, Baron D, Randuineau G, Mahè S, Cuty C, Guiguen Y (2008). Rainbow trout gonadal masculinization induced by inhibition of estrogen synthesis is more physiological than masculinization induced by androgen supplementation. Biol Reprod.

[CR85] Vizziano-Cantonnet D, Baron D, Mahe S, Cauty C, Fostier A, Guiguen Y (2008). Estrogen treatment up-regulates female genes but does not suppress all early testicular markers during rainbow trout male-to-female gonadal transdifferentiation. J Mol Endocrinol.

[CR86] Wang DS (2011) Transcriptome analysis of the molecular mechanisms for tilapia sex determination, differentiation and sex reversal. 9th Int Symp Reprod Physiol Fish, Cochin, India (abstract)

[CR87] Wang DS, Zhou LY, Kobayashi T, Matsuda M, Shibata Y, Sakai F, Nagahama Y (2010). Doublesex- and Mab-3-related transcription factor-1 repression of aromatase transcription, a possible mechanism favoring the male pathway in tilapia. Endocrinology.

[CR88] Wang XG, Bartfai R, Sleptsova-Freidrich I, Orban L (2007). The timing and extent of ‘juvenile ovary’ phase are highly variable during zebrafish testis differentiation. J Fish Biol.

[CR89] Yano A, Nicol B, Valdivia K, Juanchich A, Desvignes T, Caulier M, Vazir Zadeh A, Guerin A, Jouanno E, Nguyen T, Mourot B, Rime H, Bodinnier P, Cauty C, Quillet E, Guyomard R, Bobe J, Fostier A, Guiguen Y (2011) Sex in salmonids: from gonadal differentiation to genetic sex determination. 9th Int Symp Reprod Physiol Fish, Cochin, India (abstract)

[CR90] Zama AM, Uzumcu M (2010). Epigenetic effects of endocrine-disrupting chemicals on female reproduction: an ovarian perspective. Front Neuroendocrinol.

[CR91] Zhang Z, Wang Y, Wang S, Liu J, Warren W, Mitreva M, Walter RB (2011) Transcriptome analysis of female and male *Xiphophorus maculatus* Jp 163 A. PLoS One 6(4):e18379) 10.1371/journal.pone.0018379 PMC3071723 21483681

